# Indirect Effects of PM_2.5_ Exposure on COVID-19 Mortality in Greater Jakarta, Indonesia: An Ecological Study

**DOI:** 10.5334/aogh.4411

**Published:** 2024-05-27

**Authors:** Budi Haryanto, Indang Trihandini, Fajar Nugraha, Fitri Kurniasari

**Affiliations:** 1 Department of Environmental Health, Faculty of Public Health, Universitas Indonesia, ID; 2 Research Center for Climate Change, I-SER, Universitas Indonesia, ID; 3 Department of Biostatistics and Population Studies, Faculty of Public Health, Universitas Indonesia, ID; 4 Department of Biostatistics and Population Studies, Faculty of Public Health, Universitas Indonesia, ID

**Keywords:** Air pollution, PM_2.5_, COVID-19 mortality, Greater Jakarta cities

## Abstract

**Background:**

Air pollution, including PM_2.5_, was suggested as one of the primary contributors to COVID-19 fatalities worldwide. Jakarta, the capital city of Indonesia, was recognized as one of the ten most polluted cities globally. Additionally, the incidence of COVID-19 in Jakarta surpasses that of all other provinces in Indonesia. However, no study has investigated the correlation between PM_2.5_ concentration and COVID-19 fatality in Jakarta.

**Objective:**

To investigate the correlation between short-term and long-term exposure to PM_2.5_ and COVID-19 mortality in Greater Jakarta area.

**Methods:**

An ecological time-trend study was implemented. The data of PM_2.5_ ambient concentration obtained from Nafas Indonesia and the National Institute for Aeronautics and Space (*LAPAN*)/National Research and Innovation Agency (*BRIN*). The daily COVID-19 death data obtained from the City’s Health Office.

**Findings:**

Our study unveiled an intriguing pattern: while short-term exposure to PM_2.5_ showed a negative correlation with COVID-19 mortality, suggesting it might not be the sole factor in causing fatalities, long-term exposure demonstrated a positive correlation. This suggests that COVID-19 mortality is more strongly influenced by prolonged PM_2.5_ exposure rather than short-term exposure alone. Specifically, our regression analysis estimate that a 50 µg/m3 increase in long-term average PM_2.5_ could lead to an 11.9% rise in the COVID-19 mortality rate.

**Conclusion:**

Our research, conducted in one of the most polluted areas worldwide, offers compelling evidence regarding the influence of PM_2.5_ exposure on COVID-19 mortality rates. It emphasizes the importance of recognizing air pollution as a critical risk factor for the severity of viral respiratory infections.

## Introduction

The global battle against the COVID-19 pandemic has unveiled the intricate interplay between environmental factors and public health outcomes. Air pollution has emerged as a critical determinant affecting COVID-19 fatalities worldwide [1]. In regions such as Italy and Spain, where concentrations of nitrogen oxide (NO_2_) rank highest among European countries, the correlation between air pollution and increased fatality rates has been reported [2]. Likewise, in the United States, prolonged exposure to air pollution has been linked to a significant exacerbation of COVID-19 symptoms [1,2,3]. This global context emphasizes the need for comprehensive investigations into the impact of air quality on the outcomes of COVID-19.

Zooming into the Southeast Asian context, Jakarta, the sprawling capital city of Indonesia with a population exceeding 10.7 million as of 2020, presents a vivid illustration of the challenges posed by air pollution [4]. Despite its substantial population, the air quality in Jakarta consistently hovers at poor levels, raising concerns about potential implications for public health. In 2019, Jakarta ranked 12^th^ among the most polluted cities worldwide [5]. Among other provinces in Indonesia, Jakarta has experienced the highest incidence of COVID-19, with a total of 1,249,631 positive cases and 15,282 deaths as of May 22, 2022. Surprisingly, despite these alarming statistics, no study has systematically investigated the potential correlation between air pollution levels and the mortality rates of COVID-19 in Jakarta.

PM_2.5_ is airborne particulate matter that can be inhaled into the distal regions of the lung, with the ability to carry hazardous substances into the lung [6]. Numerous studies have now shown that the inhalation of particulate matter pollution has a strong correlation with the prevalence and mortality of COVID-19, as well as with increased susceptibility to diseases [1,2,3,7,8,9]. However, studies on the time-lags of adverse effects of PM_2.5_ exposure are still limited. Therefore, this study aimed to fill this critical gap in research by examining the association between PM_2.5_ exposure and COVID-19 mortality, both in the short-term and the long-term time lags. Conducted in one of the most air-polluted cities, the results of this study will provide insights that can contribute not only to Jakarta’s public health strategy, but also to the broader global understanding.

## Methods

**Figure 1:**
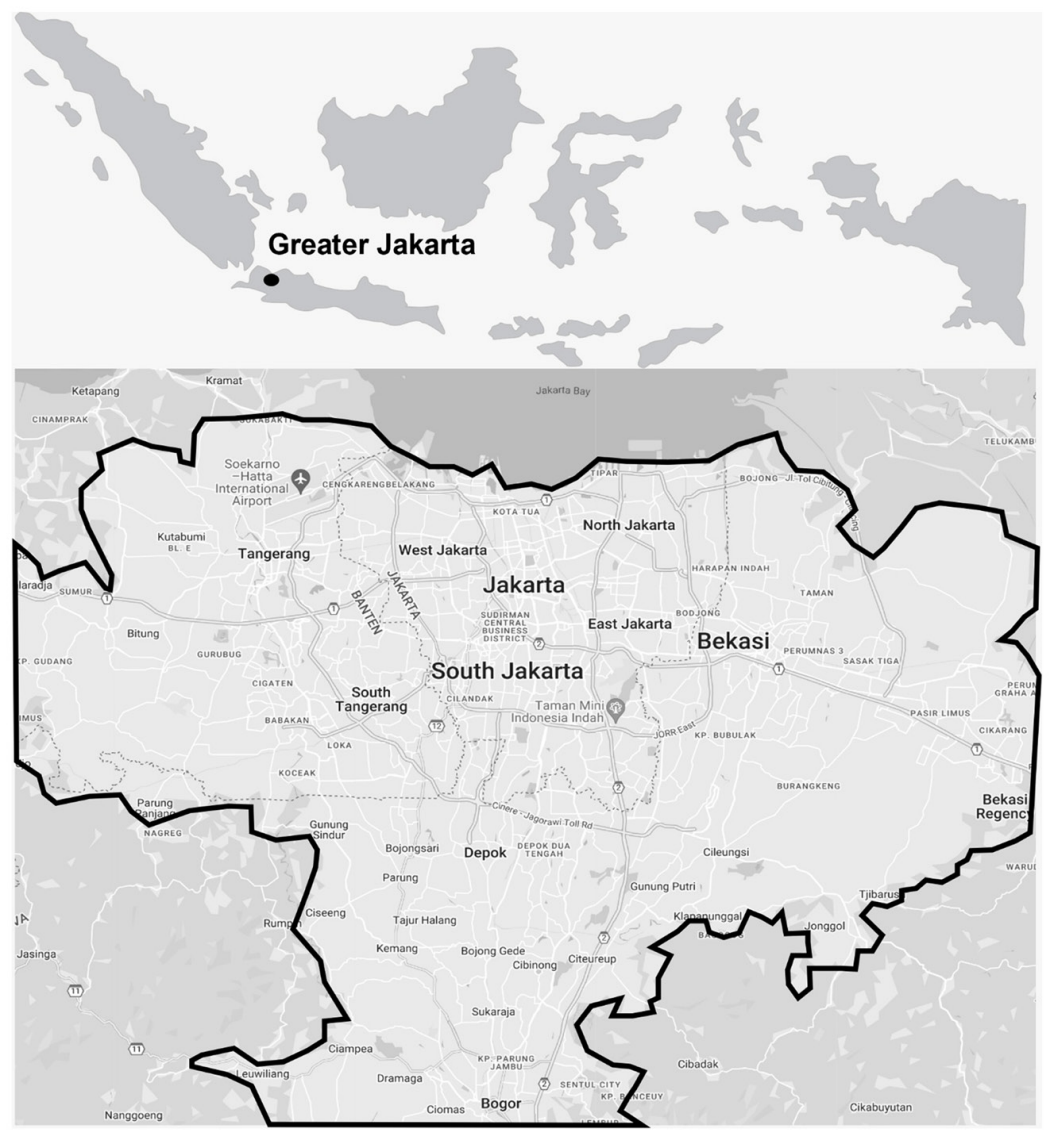
Modified maps indicating the Greater Jakarta (original source from Google Maps).

An ecological time-trend design study was implemented to analyse daily datasets for PM_2.5_ concentrations and COVID-19 mortality in the Greater Jakarta area, which consisted of 12 cities or regencies: Bekasi city, Bekasi regency, Tangerang city, South Tangerang city, Bogor city, Bogor regency, Depok city, West Jakarta, East Jakarta, North Jakarta, Central Jakarta, and South Jakarta (Figure 1). Situated between 106°33′–107° E longitude and 5° 48′ 30″–6° 10′ 30″ S latitude, the Greater Jakarta region spans approximately 652 km^2^. Characterized by a humid tropical climate, it experiences annual rainfall ranging from 1,500 to 2,500 mm, influenced by the monsoons.

Two dissimilar sources of PM_2.5_ concentration were collected for short-term and long-term exposure analyses. The daily ambient concentration of PM_2.5_ during the pandemic from March 2020 to May 2021, obtained from over one hundred low-cost monitoring stations, was provided by NafasIDN (https://nafas.co.id/) for short-term exposure analysis. Additionally, ambient PM_2.5_ concentrations from March 2016 to December 2018 were provided by the National Institute for Aeronautics and Space (LAPAN) and the National Research and Innovation Agency (BRIN) for long-term exposure analysis. The daily data of COVID-19 death for each city/regency in Greater Jakarta area were obtained from the City’s Health Office. This source presents the most comprehensive city-level COVID-19 data reported to date by the Ministry of Health and the National COVID-19 Taskforce. We collected the COVID-19 mortality data spanning from 2020 to 2021. Spearman’s correlation analysis was conducted to examine the correlation coefficient (r) values and assess statistical significance. Simultaneously, a regression model was employed to predict the association between PM_2.5_ concentration and the number of COVID-19 deaths. These statistical analyses were carried out using the SPSS statistical software.

## Findings

**Figure 2:**
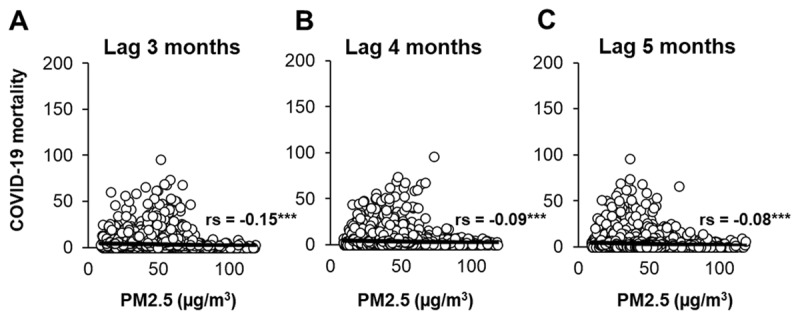
Short-term exposure of PM_2.5_ and COVID-19 deaths in different time lags. The correlation between PM_2.5_ concentration and number of new deaths during COVID-19 in lag 3 months (A), lag 4 months (B), and lag 5 months (C), as presented. Spearman’s rank correlation coefficient (rs) is presented.

Initially, we investigated the effects of short-term PM_2.5_ exposure on COVID-19 mortality across a combined 12 cities/regencies (Figure 2). Spearman correlation tests were employed to examine the lagged correlation between COVID-19 deaths and PM_2.5_ at 3 months lag (Figure 2A), 4 months lag (Figure 2B), and 5 months lag (Figure 2C). Our findings reveal a negative correlation between PM_2.5_ concentration and COVID-19 deaths across all time lags.

Significantly different values denoted as follows: ***, p < 0.001. Then, Spearman correlation tests between COVID-19 deaths and PM_2.5_ were conducted in each city or regency (Table 1). We observed a negative correlation between COVID-19 deaths and PM_2.5_ concentrations in Depok city (–0.242), West Jakarta (–0.453), East Jakarta (–0.225), North Jakarta (–0.239), Central Jakarta (–0.147), and South Jakarta (–0.163). In contrast, a significantly positive correlation between COVID-19 deaths and PM_2.5_ was found in Tangerang city (r = 0.203) and South Tangerang city (r = 0.401). We could not find any significant correlation between COVID-19 deaths and PM_2.5_ in Bekasi city, Bekasi regency, Bogor city, and Bogor regency.

**Table 1:** Correlation between short-term exposure of PM_2.5_ and COVID-19 deaths in each city or regency in Greater Jakarta.


City/Regency	Spearman correlation^a^	

	rs	P-value

Bekasi City	0.031	0.641

Bekasi Regency	–0.089	0.197

Tangerang City	**0.203**	**0.003**

South Tangerang City	**0.401**	**0.0001**

Bogor City	–0.052	0.439

Bogor Regency	0.032	0.637

Depok City	**–0.242**	**0.0001**

West Jakarta	**–0.453**	**0.0001**

East Jakarta	**–0.225**	**0.0001**

North Jakarta	**–0.239**	**0.0001**

Central Jakarta	**–0.174**	**0.0001**

South Jakarta	**–0.163**	**0.0001**


^a^ Spearman correlation test on 5 months lag.

**Figure 3:**
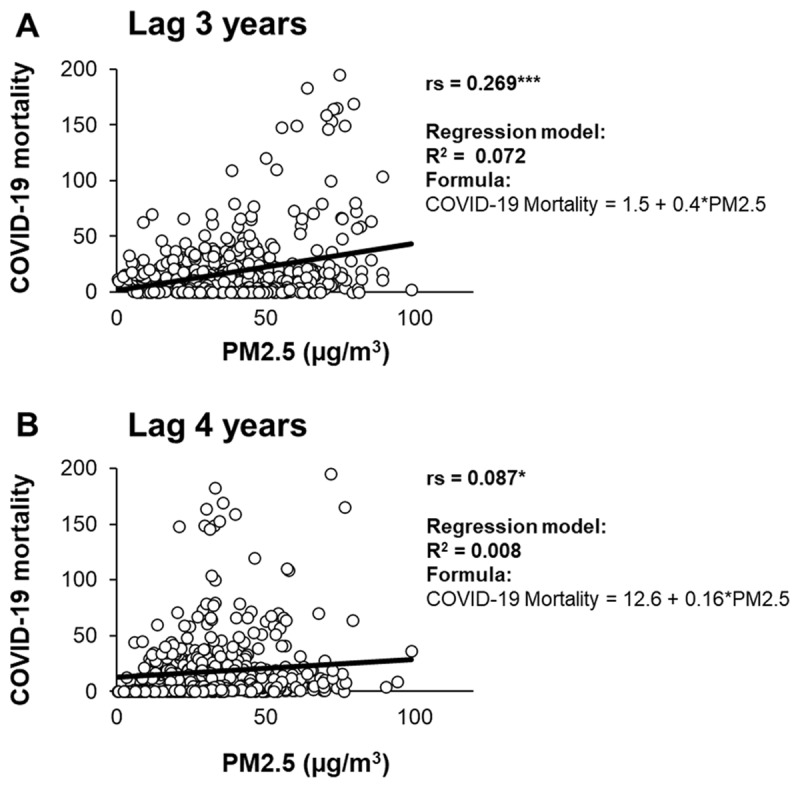
Long-term exposure of PM_2.5_ and COVID-19 deaths. The correlation between PM_2.5_ concentration and the number of new deaths during COVID-19 is examined with lags of 3 years (A) and 4 years (B). Spearman’s rank correlation coefficient (rs) and the coefficient of determination for regression (R^2^) are presented. Significantly different values denoted as follows: *, p < 0.05; ***, p < 0.001.

The effects of long-term PM_2.5_ exposure on COVID-19 mortalities further investigated with lags of 3 years (Figure 3A) and 4 years (Figure 3B). The results showed a significantly positive correlation between PM_2.5_ and COVID-19 deaths in both lag 3 years (r = 0.269) and lag 4 years (r = 0.087). The linear regression model demonstrated the formula of COVID-19 mortality = 1.5 + 0.4*PM_2.5_ for lag 3 years and COVID-19 mortality = 12.6 + 0.16*PM_2.5_ for lag 4 years.

## Discussion

In this study, we investigated the correlation between both short-term and long-term exposure to PM_2.5_ and COVID-19 deaths. Despite reports from various countries indicating a decrease in air pollution during the COVID-19 pandemic associated with lockdown restrictions [10,11], our investigation revealed that the annual mean levels of PM_2.5_ in each city/regency in the Greater Jakarta area remained higher than the WHO guideline values of 5 µg/m^3^ (Supl. Fig. 1). This indicates the persistence of severe air pollution in Greater Jakarta even during the pandemic period.

Firstly, considering the delayed effects of PM_2.5_ on mortality reported in a previous study [12], we conducted an investigation on the short-term exposure of PM_2.5_ on COVID-19 deaths with lags of 3 months, 4 months, and 5 months (Figure 2). The analysis identified a negative correlation between PM_2.5_ and COVID-19 deaths across the combined 12 cities/regencies in the Greater Jakarta area at all time lags. However, a positive correlation was observed in Tangerang city and South Tangerang city which have been reported as highly impacted by air pollution [13] and demonstrated as having one of the highest weekly increases in positive COVID-19 cases [14]. While evidence regarding the association between short-term PM_2.5_ exposure and mortality remains contradictory [15], several studies suggest that exposure PM_2.5_ may not significantly impact COVID-19 severity and mortality in the short term [9,16]. However, instead of directly increasing COVID-19 mortality rates, most studies have found a strong correlation between short-term PM_2.5_ exposure and an increase in COVID-19 incidence in many countries, including United States [17], Iran [18], and Malaysia [19]. The rise in COVID-19 cases is attributed to PM_2.5_, which can potentially carry the SARS-CoV-2 virus [20], aiding its transmission into the respiratory regions of the human lung and the dissemination of pollutants throughout the systemic circulation [12,21]. Additionally, PM_2.5_ could also reduce mucociliary clearance function which could increase the susceptibility and severity of COVID-19 [2]. Despite reports indicating that acute exposure to PM_2.5_ could exacerbate the severity of COVID-19 patients [22], our findings suggest that it may not be sufficient to cause fatalities.

Following the discovery that the immediate adverse effects of PM_2.5_ exposure on COVID-19 mortality are not apparent within the short term, we extended our investigation to analyse long-term exposure data with lags of 3 years and 4 years (Figure 3). The PM_2.5_ concentration data from 2016 to 2018 utilized for the analysis (Suppl. Fig. 2). Using the data from the National Institute for Aeronautics and Space (*LAPAN*) and the National Research and Innovation Agency (BRIN), the monthly average concentrations of PM_2.5_ in 2016–2018 consistently remained higher than WHO guideline values, indicating chronic exposure to unhealthy air quality among the residents in the Greater Jakarta area. Our Spearman test demonstrated a positive correlation between PM_2.5_ levels and COVID-19 deaths in both time lags of 3 years and 4 years. Similar results have been identified in previously published studies, indicating that long-term exposure to air pollutants exceeding WHO guidelines could potentially worsen not only the severity [23], but also the mortality rates of COVID-19 [2,3].

Finally, our regression model suggested that the increase in long-term PM_2.5_ concentration followed by an increase in COVID-19 deaths in the Greater Jakarta area (Figure 3). It is predicted that increase of 50 µg/m^3^ in long-term average PM_2.5_ is associated with an 11.9% increase in the COVID-19 mortality rate. Similar trends have been reported in several previous studies. A study in China found that a 10 μg/m3 increase is associated with a 0.24% increase in the daily COVID-19 case fatality rate [24]. Additionally, it has been reported that a 1 μg/m3 increase in PM_2.5_ exposure resulted in nearly a 10% increase in COVID-19 mortality in the United States [25], a 1.3% increase in COVID-19 mortality in Chile [26], and a 9% increase in COVID-19 mortality in North Italy [27]. The increased mortality rates associated with COVID-19 due to long-term exposure to PM_2.5_ may be attributed to the potential systemic damage induced by PM_2.5_, resulting in the generation of oxidative stress and inflammation [7]. Individuals chronically exposed to higher levels of PM_2.5_ pollution are more susceptible to developing cardiopulmonary diseases, including ischemic heart disease [28], chronic obstructive pulmonary disease [29], lung cancer [30], and strokes [31]. These adverse health outcomes may exacerbate the effects of COVID-19, leading to fatalities. Additionally, PM_2.5_ is known to upregulate ACE-2 expression [32,33], which is believed to exacerbate the severity of COVID-19 by facilitating SARS-CoV-2 viral adhesion to systemic cells [6]. Thus, the findings in this study reinforce the discovery that COVID-19 mortality is primarily influenced by longer-term rather than short-term factors.

## Limitation of the Study

While this study holds the potential to significantly contribute to the evidence, we have acknowledged several key limitations. Firstly, our reliance on an ecological study design and administrative databases limited the availability of personal information, which was confined to age, sex, primary diagnosis, and place of residence. Additionally, since the data were analysed in aggregate, this design hindered our ability to investigate whether the population under study remained in the same geographic area or relocated randomly. Secondly, our study employed relatively simplistic statistical methods, such as the Spearman correlation. Despite their simplicity, these methods still provide valuable insights into the correlation trends between PM_2.5_ exposure and COVID-19. Thirdly, due to limitations in the data source, we were unable to investigate various meteorological factors that influence pollution levels, including temperature, precipitation, wind speed, and precipitation, as well as the influence of other pollutants in this study. Therefore, further epidemiological research with adequate consideration of confounding factors is necessary to confirm the results and thoroughly examine the association between PM_2.5_ exposure and COVID-19 mortality. However, as evidence regarding PM_2.5_ exposure and COVID-19 mortality in Southeast Asia, particularly in Indonesia, remains limited, our current study still offers valuable evidence on this issue.

## Conclusion

In this study, we investigated the association between both short-term and long-term exposure to PM_2.5_ and the number of COVID-19 deaths using an ecological study design. The findings revealed that COVID-19 mortality is primarily influenced by longer-term exposure rather than short-term exposure to PM_2.5_. Conducted in one of the locations with the highest levels of air pollution globally, our study provides clearer evidence of the impact of PM_2.5_ exposure on COVID-19 mortality. Although COVID-19 has not been classified as a pandemic since 2023, the results of this study remain relevant for predicting the effects of air pollution and other mortality associated with viral respiratory infections.

## Additional File

The additional file for this article can be found as follows:

10.5334/aogh.4411.s1 Supplementary Material. Suppl. Fig. 1–Suppl. Fig. 2.
