# Supplementary material for: Indirect Effects of PM2.5 Exposure on COVID-19 Mortality in Greater Jakarta, Indonesia: An Ecological Study
Source: Ann Glob Health. 2024 May 27;90(1):34. doi: 10.5334/aogh.4411 (PMC11141510; doi:10.5334/aogh.4411)
Supplement: Supplementary Material. — Suppl. Fig. 1–Suppl. Fig. 2. [file agh-90-1-4411-s1.pdf]

Supplementary Material

Indirect Effects of $PM_{2.5}$ Exposure on COVID-19 Mortality in Greater Jakarta, Indonesia: An Ecological Study

Budi Haryanto[1 3]*, Indang Trihandini[2], Fajar Nugraha[2], Fitri Kurniasari[1]

[1]Department of Environmental Health, Faculty of Public Health, Universitas Indonesia

[2]Department of Biostatistics and Population Studies, Faculty of Public Health, Universitas Indonesia

[3]Research Center for Climate Change, I-SER, Universitas Indonesia

***Corresponding author: Prof. Budi Haryanto**

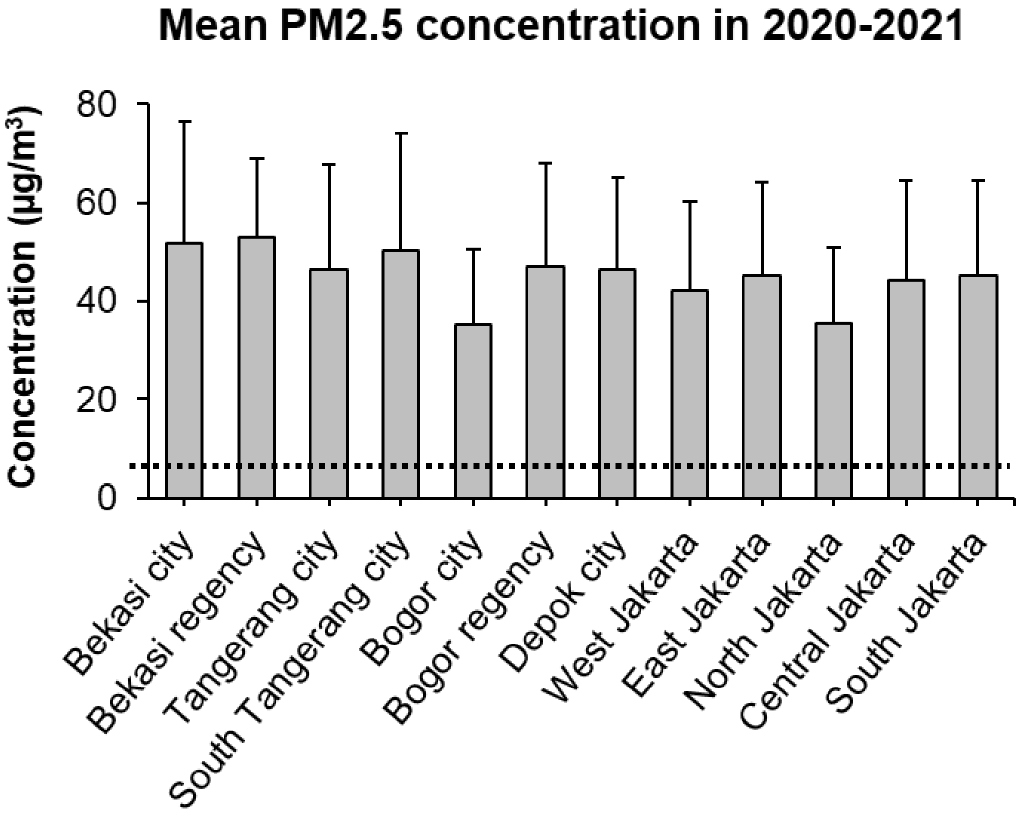

Suppl. Fig. 1. Annual mean PM2.5 concentration during the pandemic. Provided by *Nafas IDN* application, the mean ± SD concentration of PM2.5 between 2020–2021 in each city and regency in Greater Jakarta, is presented. The dotted line indicates the WHO guidelines for annual PM2.5 concentration, which is 5 μg/m3.

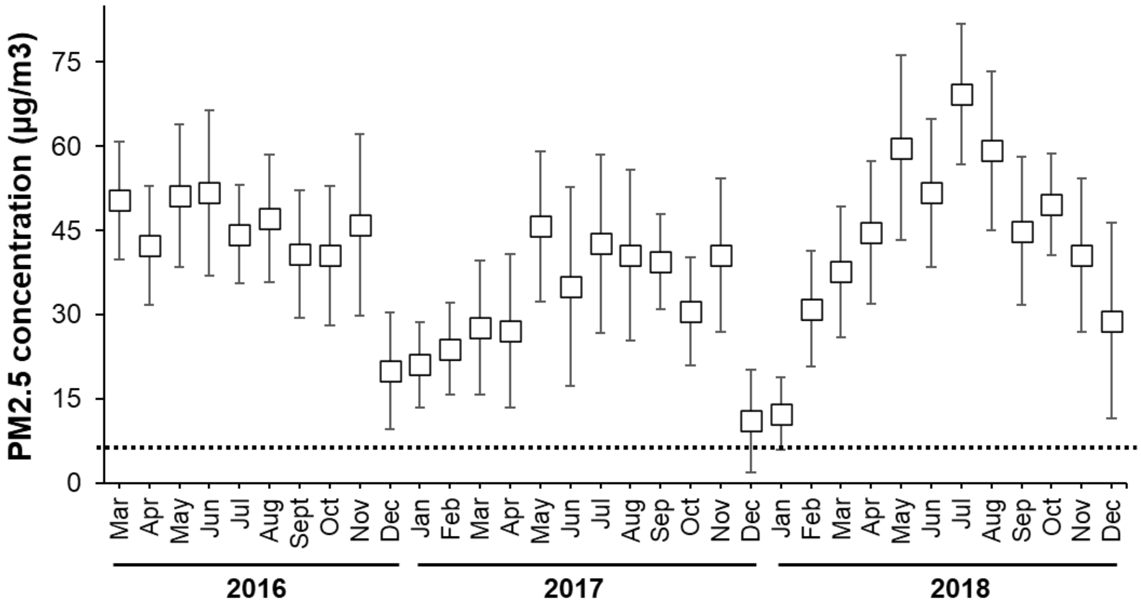

Suppl. Fig.2. Monthly average concentration of PM2.5 in Jakarta Greater Area from 2016–2018. Provided by National Institute for Aeronautics and Space (LAPAN)/National Research and Innovation Agency (BRIN), the mean ± SD concentration of PM2.5 between 2016–2018 in each city and regency in Greater Jakarta, is presented. The dotted line indicates the WHO guidelines for annual PM2.5 concentration, which is 5 µg/m3.
